# Physical Activity Impacts Walking Distances and Energy Consumption of Patients with Peripheral Artery Disease

**DOI:** 10.1093/geroni/igab046.3255

**Published:** 2021-12-17

**Authors:** Nathaniel Evans, Mahdi Hassan, Danae Dinkel, Jason Johanning, Iraklis Pipinos, Sara Myers

**Affiliations:** 1 University of Nebraska Omaha, Omaha, Nebraska, United States; 2 University of Nebraska at Omaha, Omaha, Nebraska, United States; 3 University of Nebraska Medical Center, Omaha, Nebraska, United States

## Abstract

Lower extremity peripheral artery disease (PAD) is attributed to buildup of atherosclerotic plaques preventing adequate blood flow, leading to pain during walking, and ultimately physical inactivity. Normal day-to-day levels of physical activity may impact the distance a subject can walk before claudication pain onset, as well as their energy consumption capabilities. This study compared walking performance (initial claudication distance (ICD) and absolute claudication distance (ACD)), and energy consumption (EC) between active and inactive subjects with PAD. The distinction between groups was made using previous research that declared the average PAD patient walks 3586 steps/day. Ten subjects were classified as active (□3586 average steps/day) and sixteen participants as inactive (<3586 steps/day) based on a 7-day accelerometer measurement. The Gardner progressive treadmill test was used to asses ICD, ACD, and EC. EC was measured using a metabolic cart and calculated from the second minute of walking and the last minute prior to stopping due to claudication pain. The average ICD and ACD for the active group were 130.6±106.7 meters and 306.0±184.7 meters, respectively and 143.8±119.0 meters and 248.0±156.0 meters, respectively for the inactive group. The average EC for the second minute and last minute were 9.6±1.9 mlkg-1min-1 and 11.5±2.4 mlkg-1min-1 respectively for active group and 7.0±3.1 mlkg-1min-1 and 8.1±3.8 mlkg-1min-1 respectively for inactive group. The data suggests that the active group had better walking performance and greater energy consumption indicating increased efficiency of oxygen transport and extraction capability in the leg muscles.

